# From patient care to research: a validation study examining the factors contributing to data quality in a primary care electronic medical record database

**DOI:** 10.1186/s12875-015-0223-z

**Published:** 2015-02-05

**Authors:** Nathan Coleman, Gayle Halas, William Peeler, Natalie Casaclang, Tyler Williamson, Alan Katz

**Affiliations:** Department of Family Medicine, University of Manitoba, Winnipeg, MB Canada; Department of Community Health Sciences, University of Calgary, Calgary, AB Canada; Department of Community Health Sciences, Manitoba Centre for Health Policy, University of Manitoba, 408-727 McDermot Ave, Winnipeg, MB R3E 3P5 Canada

**Keywords:** Electronic Medical Records, Primary Care, Chronic Disease, Health Information Systems

## Abstract

**Background:**

Electronic Medical Records (EMRs) are increasingly used in the provision of primary care and have been compiled into databases which can be utilized for surveillance, research and informing practice. The primary purpose of these records is for the provision of individual patient care; validation and examination of underlying limitations is crucial for use for research and data quality improvement. This study examines and describes the validity of chronic disease case definition algorithms and factors affecting data quality in a primary care EMR database.

**Methods:**

A retrospective chart audit of an age stratified random sample was used to validate and examine diagnostic algorithms applied to EMR data from the Manitoba Primary Care Research Network (MaPCReN), part of the Canadian Primary Care Sentinel Surveillance Network (CPCSSN). The presence of diabetes, hypertension, depression, osteoarthritis and chronic obstructive pulmonary disease (COPD) was determined by review of the medical record and compared to algorithm identified cases to identify discrepancies and describe the underlying contributing factors.

**Results:**

The algorithm for diabetes had high sensitivity, specificity and positive predictive value (PPV) with all scores being over 90%. Specificities of the algorithms were greater than 90% for all conditions except for hypertension at 79.2%. The largest deficits in algorithm performance included poor PPV for COPD at 36.7% and limited sensitivity for COPD, depression and osteoarthritis at 72.0%, 73.3% and 63.2% respectively. Main sources of discrepancy included missing coding, alternative coding, inappropriate diagnosis detection based on medications used for alternate indications, inappropriate exclusion due to comorbidity and loss of data.

**Conclusions:**

Comparison to medical chart review shows that at MaPCReN the CPCSSN case finding algorithms are valid with a few limitations. This study provides the basis for the validated data to be utilized for research and informs users of its limitations. Analysis of underlying discrepancies provides the ability to improve algorithm performance and facilitate improved data quality.

## Background

Electronic Medical Records (EMRs) are increasingly used in the provision of primary care, recording pertinent clinical data with the promise of improved efficiency, quality of care and patient safety [1]. Data in EMRs is also extracted and compiled into databases which provide a valuable resource for primary care research and surveillance [2]. EMR data contains comprehensive records of diagnoses, visits, laboratory tests, prescriptions and physical examination findings and therefore has several advantages over other sources of data, such as administrative databases and patient or visit-level surveys [3]. However, many factors can influence EMR data quality. As these databases are increasingly used for surveillance, research and informing practice, the process of evaluating and maintaining data quality is of paramount importance [4].

Data quality must be assessed within the context of its intended purpose so it must be assessed for its fitness for each particular use [5,6]. One of the great challenges of using EMR data in research is that EMR data is generally collected for the purpose of providing individual patient care and administrative purposes rather than specifically for research or surveillance [7,8]. This means that before using the data for surveillance, research or other purposes it must be validated. Furthermore, there is a need to understand the factors that affect data quality.

### The CPCSSN database

The Canadian Primary Care Sentinel Surveillance Network (CPCSSN) is a database built from EMR extracted data for the surveillance of eight chronic diseases including hypertension (HTN), diabetes mellitus (DM), osteoarthritis (OA), depression and chronic obstructive pulmonary disease (COPD). The data is compiled from 10 practice based research networks (PBRNs) across Canada [4,9].

**Figure 1:**
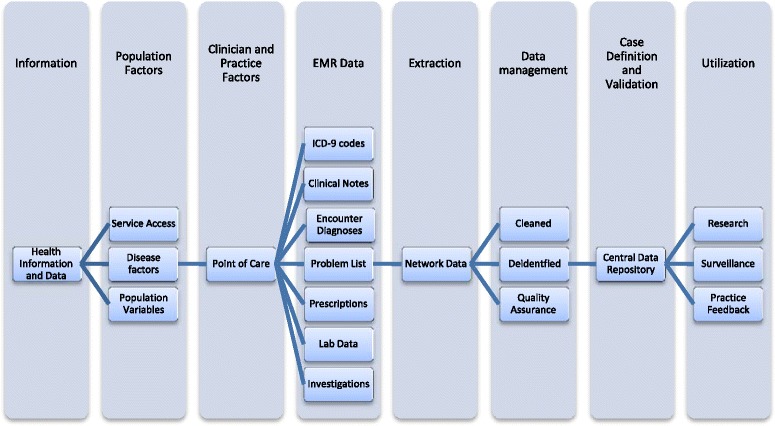
**The CPCSSN information pathway and factors affecting data quality and validity.** This figure illustrates the information pathway in CPCSSN and is based on data flow described by Kadhim-Saleh, et al. [9] and Birtwhistle, et al. [4]. Eight categories are depicted, each representing stages along a continuum beginning with health information presented by individuals, collected by practitioners at the point of care, and entered into the EMR. The information is then extracted into a regional data structure where it is cleaned and de-identified. Case definitions are applied to this regional network data prior to storage within the central data repository. The data is then ready to be utilized for surveillance, research and practice feedback. Together this illustrates the possible points where data quality can be affected.

The information pathway shown in Figure 1 illustrates how information is processed and transferred as it is presented by patients, collected by clinical practices, entered into the EMR and, after appropriate data management, stored in the central repository. Case finding algorithms are applied to the raw data within the repository, thus enabling surveillance, research and feedback reports to the participating practices [4]. At different points in this information pathway, there are potential threats to data quality.

Validation of the CPCSSN case finding algorithms has been carried out across the network [9,10]. Previously identified challenges affecting data quality include missing data, variation in terminology and misclassification of coding [4] as well as significant variation between diseases [9]. However, the rate at which these occur and the degree to which each factor contributes to overall data quality and validity has not been reported. While validation delineating measures such as positive predictive value (PPV) or sensitivity remain important [5], further delineation of the particular factors that contribute to those measures is critical to understand the context of the data [8] and the purposes for which it could be utilized. For example, a case finding algorithm with high sensitivity and good positive predictive value that identifies cases with COPD but over excludes those who also have comorbid asthma would not be fit for use in studies investigating overlap or co-occurrence of both conditions. This requires a closer examination of the underlying factors that affect algorithm performance. This study examines and describes the factors that affect data quality which impact validity of case finding algorithms in a primary care EMR database.

## Methods

We conducted a retrospective chart review of EMR data from one PBRN, the Manitoba Primary Care Research Network (MaPCReN), to characterize the various factors contributing to the validity of diagnoses in the database. Validation of the CPCSSN 2011 diagnostic algorithms was performed using the chart review as the gold standard. Chart review was selected as it has the advantages of ensuring completeness and does not depend on the cooperation of physicians or office staff [3] and is used widely in research therefore fulfilling the fitness for use criteria [5].

### Sampling

An age stratified random sample of 403 patients was taken from patients who attended one of three clinics within MaPCReN; 90% of the sample were 60 years of age and over and all of whom had at least one of the five conditions of interest according to current CPCSSN algorithms. The sample size of 400 patients was chosen to be consistent with other validation work done within CPCSSN [9,10]. This sample size calculation was determined based on the least prevalent condition, COPD, in order to target a margin of error of less than 20% per network and 10% overall for the estimated sensitivity. Chart abstractors were blinded to the algorithm that identified the diagnoses as the case finding algorithms were not applied until initial chart review was complete. Research ethics approval for the project was obtained through the Health Research Ethics Board at the University of Manitoba.

### Data collection

The review of 403 charts was carried out by two trained medical data extractors (NCo,NCa) to determine the presence or absence of the five chronic conditions of interest. They examined the entire electronic medical record to conclude whether a patient had one or more of the five conditions. The patient’s clinical information from the EMR was collected in a standardized abstraction form. Any encounters or diagnoses recorded in the chart after December 31, 2011 were excluded. Cases with any diagnostic uncertainty were reviewed with an expert clinician researcher and standardized approaches to recurring problems were developed. There were eight uncertain cases after the consultation process. These were treated as negative for purposes of computing validation statistics. Chart reviewers independently reviewed the same 150 cases to assess inter-rater reliability. After the chart review and cases with uncertainty had been reviewed, the 2011 CPCSSN algorithms were applied to the data in order to determine which cases the algorithms identified and where these differed from chart review.

### Data analysis

All statistics were calculated using SPSS Version 22 (SPSS IBM, New York, U.S.A). Sensitivity, specificity and positive predictive values of each of the case detection algorithms were calculated using chart review as the gold standard. The 95% confidence intervals were calculated using the Wilson score method [11]. Overall agreement was assessed using Cohen’s kappa. Inter-rater reliability of chart reviewers was assessed by calculating both percentage agreement for each diagnosis and cumulative kappa statistic. Discordant cases were then identified and clinical information recorded in the reviewers’ database was examined in relation to CPCSSN disease definitions to determine the probable source of discrepancy. These observations were then categorized, documented and frequency of each factor was determined.

## Results

The mean age of the patients whose charts were reviewed was 73 years and 67% were women. Ninety seven percent had at least 1 of the 5 chronic conditions of interest according to the review of the medical record. Table 1 provides details of the population demographics.

**Table 1:** **Demographics of Sample from the Manitoba Primary Care Research Network (MaPCREN)**

**Demographics**	**n (%)**
Male	132 (33)
Female	271 (67)
Age in years	
≥60	363 (90)
<60	40 (10)
**Number of chronic conditions**	
At least 1 of 5	392 (97)
2	158 (39)
3	64 (16)
4	16 (4)
5	1 (0.2)

In the sample of 150 cases that were reviewed independently by both data extractors, the percentages of agreement between them were 95% (Depression), 92% (HTN), 87% (DM), 88% (COPD), and 67% (OA) with a cumulative kappa of 0.902 (95% CI 0.87- 0.93). Sensitivity, specificity, and positive predictive values were calculated for each condition using chart review as the reference standard (Table 2).

**Table 2:** **Validation results for the CPCSSN case finding algorithms of 5 chronic conditions**

**Condition**	**CPCSSN+ Chart+**	**CPCSSN+ Chart-**	**CPCSSN- Chart+**	**CPCSSN- Chart-**	**Sensitivity**	**Specificity**	**PPV**	**Kappa**
					**% (95% CI)**	**% (95% CI)**	**% (95% CI)**	**k (95% CI)**
COPD	18	31	7	347	72.0 (52.4-85.7)	91.8 (88.6-94.2)	36.7 (24.7-50.7)	0.44 (.30-.59)
Depression	85	13	31	274	73.3 (64.6-80.5)	95.5 (92.4-97.3)	86.7 (78.6-92.1)	0.72 (.64-.80)
Diabetes	93	9	10	291	90.3 (83.0-94.6)	97.0 (94.4-98.4)	91.2 (84.1-95.3)	0.88 (.82-.93)
Hypertension	283	22	14	84	95.3 (92.2-97.2)	79.2 (70.6-85.9)	92.8 (89.3-95.2)	0.76 (.69-.84)
Osteoarthritis	180	7	105	111	63.2 (57.4-68.5)	94.1 (88.3-97.1)	96.3 (92.5-98.2)	0.46 (.39-.54)

COPD: Chronic Obstructive Pulmonary Disease; CI: Confidence Interval.

Diagnosis identified is denoted by “+”; Diagnosis not identified is denoted by “-”.

The algorithm for diabetes had high sensitivity, specificity and PPV with all scores being over 90%. The algorithm for hypertension was the only one with specificity below 90%. The largest deficits in algorithm performance include poor PPV for COPD at 37% and limited sensitivity for COPD, depression and osteoarthritis at 72%, 73% and 63% respectively.

Among the 403 charts sampled, there were a total of 249 non-matching diagnoses of all five conditions. Of these, 82 were diagnoses that were not identified by chart review but were inaccurately detected by case finding algorithms and 167 were diagnoses that were identified by chart review but undetected by algorithms. Tables 3 and 4 outline the frequency of specific observations regarding discordant cases.

**Table 3:** **Sources of discordance for diagnoses detected by case finding algorithms but not chart review**

**Category Specific factor**	**COPD**	**Depression**	**DM**	**HTN**	**OA**	**Total**
	**n (%)**	**n (%)**	**n (%)**	**n (%)**	**n (%)**	**n (%)**
**ICD-9 codes present without diagnosis**	24(77)	6(46)	8(89)	19(86)	7(100)	64(78)
Used for unrelated visit	1(3)	3(23)	5(56)	12(55)	2(29)	23(28)
Used for visit for a related condition	3(10)	-	1(11)	3(14)	1(14)	8(10)
Used for visits with queried unconfirmed diagnosis	1(3)	3(23)	2(22)	4(18)	4(57)	14(17)
Inappropriate COPD codes at single site	19(61)	-	-	-	-	19(23)
**Medications/Lab**						
Medications prescribed for other indications	8(26)	7(54)	-	3(14)	-	18(22)
Aberrant Labs	-	-	1(11)	-	-	1(1)
Total Discordant Cases (Chart -/CPCSSN +)*	31(100)	13(100)	9(100)	22(100)	7(100)	82(100)

COPD: Chronic Obstructive Pulmonary Disease; DM: Diabetes Mellitus; HTN: Hypertension; OA: Osteoarthritis.

*Total is greater than row values due to multiple factors contributing to discordance.

**Table 4:** **Sources of discordance for diagnoses detected by chart review but not case finding algorithms**

**Category Specific factor**	**COPD**	**Depression**	**DM**	**HTN**	**OA**	**Total**
	**n (%)**	**n (%)**	**n (%)**	**n(%)**	**n (%)**	**n (%)**
**Coding**						
Diagnosis not coded due to multi-problem visit	-	1(3)	-	1(7)	13(12)	15(9)
Use of non-specific ICD-9 code	-	-	-	-	13(10)	13(8)
Formatting (e.g. missing decimal place)	-	-	-	-	4(4)	4(2)
**Terminology**						
Alternate terminology used	-	-	-	1(7)	17(16)	18(11)
**Access Limitations**						
Diagnosis record limited to free text fields	3(43)	15(48)	4(40)	6(43)	47(45)	75(45)
Diagnosis confirmed from inaccessible investigations or documents	2(29)	-	-	2(14)	14(13)	18(11)
Not detected from problem list	-	1(3)	-	-	2(2)	3(1)
Data missing after extraction/cleaning	-	6(19)	1(10)	-	30(29)	37(22)
**Case Definition**						
Indicators excluded due to co-existing condition	2(29)	7(23)	-	4(29)	-	13(8)
Insufficient frequency of indicator (e.g. limited visits or labs)	-	-	5(50)	2(14)	-	7(4)
**Medications**						
Medication not included in case definition	1(14)	9(29)	-	7(50)	-	17(10)
**Validation Factors**						
Uncertainty of presence of diagnosis after abstraction	-	-	1(10)	-	-	1(1)
Total Discordant Cases (Chart Review +/CPCSSN-)*	7(100)	31(100)	10(100)	14(100)	105(100)	167(100)

COPD: Chronic Obstructive Pulmonary Disease; DM: Diabetes Mellitus; HTN: Hypertension; OA: Osteoarthritis.

*Total is greater than row values due to multiple factors contributing to discordance.

### Discordance analysis

#### Diagnoses detected by algorithms; not found on chart review

Seventy eight percent of diagnoses detected by algorithms but not found on chart review were due to the presence of ICD-9 codes without a diagnosis documented in the medical record. Some ICD-9 codes were used for visits for related or similar conditions. For example, reactive airway disease was coded as COPD, prediabetes was coded as diabetes, white coat hypertension was coded as hypertension and gout was coded as osteoarthritis. ICD-9 codes were also used for visits where a diagnosis was considered but was unconfirmed or later ruled out. Other examples of inaccurate diagnostic codes include the use of diabetes codes for routine lab work which included screening for diabetes and the use of a depression code in a visit where a patient was recorded as talking about her husband’s depression.

For COPD, most inaccurate ICD-9 codes were from 19 cases occurring at a single site where ICD-9 billing codes for COPD were incorrectly used for visits with no relation to COPD. The vast majority of these occurred within one year and likely reflect a small number of users at that site.

Twenty two percent of diagnoses inaccurately detected were due to medications used for other indications. Examples include patients identified by the algorithm as having COPD with medications prescribed for acute bronchitis, chronic cough and asthma. In five instances, depression was inappropriately detected due to antidepressant medication used for pain, headaches or anxiety. In three cases patients given a nasal preparation of ipratropium for rhinitis were identified as having COPD.

#### Diagnoses from chart review; undetected by algorithms

The factors leading to 167 diagnoses found on chart review that were not identified by the CPCSSN algorithms were more varied. The most common omission occurred due to the diagnosis being recorded in the free text only. Over 20% of the diagnoses recorded only in the free text were due to multiple complaint visits where only one diagnosis was recorded and coded. Some diagnoses were only recorded or confirmed in investigations or documents not accessible to algorithms including pulmonary functions tests for COPD, 24 hour blood pressure monitoring reports for hypertension, x-ray findings for OA and letters from consultants. The review revealed data discrepancies that occurred during the data extraction and cleaning process resulting in 36 undetected diagnoses. For example, one patient with diabetes was not detected due to missing lab values which confirmed the diagnosis.

Comorbid conditions also contributed to inappropriate exclusion. Certain fields of data were missed by the algorithm if two or more diagnoses used the same medications. Inappropriate exclusion based on comorbidity was observed in two patients who had both asthma and COPD, seven patients with both anxiety and depression, and four hypertensive patients with kidney stones, congestive heart failure or diabetes.

There were 105 unidentified diagnoses of osteoarthritis which were a result of additional factors, which included the use of alternate less specific coding, such as arthritis not otherwise specified or knee pain, or alternate labelling, such as “overuse arthritis.” Two cases had the osteoarthritis diagnosis recorded in the problem list but was not detected by algorithms, leaving us to postulate that alternate labeling resulted in the omissions.

## Discussion

From the examination of algorithm performance and contributing factors affecting the observed discrepancies, this study provides insight into the challenges of attaining and maintaining quality data in primary care EMR databases. The excellent performance of the diabetes algorithm [9] may be attributed to its breadth, which includes recorded diagnoses, ICD-9 codes, highly specific medications and laboratory values thereby enabling greater levels of agreement. However, the PPV of only 37% of the COPD algorithm is significantly lower than those observed in other primary care EMR databases [9,12]. Limitations in sensitivity have been observed previously [9], however our examination of underlying factors provides insight into the causes as well as possible solutions. The framework presented in Figure 1 encompasses the factors contributing to the algorithm limitations evident in our investigations with major contributors being clinical practice factors, EMR data limitations and data management.

### Clinician and practice factors

Practice based factors have been found to contribute the largest variation in data quality [6,13,14]. In our study, 45% of all diagnoses missed by the algorithms were due to diagnoses being recorded solely in the free text fields and without use of an ICD-9 code or listing in the problem list. The variation between practices is evident in the example of COPD, where the largest source of discrepancy of inappropriate ICD-9 codes occurred at a single site. Physician ICD-9 coding accuracy has been previously shown to be adversely affected by higher workload, clinician uncertainty, patient complexity, and the possible stigma associated with certain conditions [14,15]. Nonetheless, practice-based factors can be addressed through measures such as effective training and communication with both clinical and office staff at each practice site [4,16]. Additionally, the use of data quality measures or feedback to the practice has been shown to improve recording practices [17]. This can be further improved by providing incentives for good quality coding and records [5].

Alternatively, computer based interventions, such as natural language processing and algorithms used to access free text fields could improve detection [18]. However, this may also cause new errors based on detection of suspected or possible cases rather than confirmed conditions [18]. Widely variable terminology use by different clinicians makes this particularly challenging. In our study, over 15 different terms were observed for osteoarthritis.

### Challenges unique to primary care EMR data

Primary care EMR data presents unique challenges in attaining accurate case detection. In primary care, undifferentiated illness is common and the diagnostic process often involves a degree of uncertainty and significant complexity [19,20]. Recorded ICD-9 codes are intended to identify or label a visit in terms of a diagnosis. However, the diagnostic process may involve multiple visits and physicians provide a code that best reflects the status of an investigation before the diagnostic endpoint is reached. Consequently, cases where a physician’s diagnosis is tentative may be classified as a definitive diagnosis [9], as was observed in 17% of inappropriately identified diagnoses. Our study also found physicians using less specific codes which resulted in 10% of unidentified osteoarthritis diagnoses being missed. Previous studies have found that ICD9 codes often do not reflect the dominant or most time consuming aspect of a primary care visit [20]. In part this may be due to multiple comorbidities and multi-problem visits, which played a role in 17% of unidentified cases. Co-morbidity is a common occurrence in primary care and our data indicate 39% of the sample with at least two chronic conditions and a potentially higher percentage if acute problems are considered.

While prescription data appears to be well recorded in primary care EMRs [21], it’s utilization for case detection remains challenging. Our study found that 54% of depression diagnoses that were inappropriately identified were due to medications prescribed for other indications. Studies have shown that anti-depressants are increasingly being used for many non-psychiatric conditions in primary care [22,23]. Thus, determining which medications are specific enough to be used for case definitions is difficult in primary care as indications for a given medication change over time and may vary among practitioners. Additionally, detailed information such as route of administration is important in some conditions, such as COPD, where nearly half of the prescriptions causing the diagnosis to be inappropriately detected were due to nasal drug prescriptions. Maintaining up to date medication lists and appropriate exclusion criteria is therefore crucial.

### Disease specific case definition challenges

Comprehensive yet discriminating case definitions and accurate case finding algorithms are key components of data quality [24]. However, there are challenges to identifying cases that truly reflect the diagnosis of interest rather than clinically insignificant findings. For example, 80% of individuals over the age of 50 have radiographic evidence of osteoarthritis [25] yet there is poor association between radiographic findings of osteoarthritis and clinical symptoms [26]. For chart reviewers in this study, isolated radiologic evidence was not considered sufficient to label the diagnosis; a recorded diagnosis or treatment for osteoarthritis by the physician was also required for reviewers to identify the condition.

### Future research: algorithm modifications and testing

With 22% of undetected cases occurring as a result of absent billing fields after data extraction and cleaning processes, this research underscores the importance of in-depth data validation and quality assessments by skilled staff. [8] More importantly, the findings in this study suggest a number of limitations in the algorithms that may be amenable to changes and future testing. A number of the factors identified can be modified through data cleaning, data restoration and algorithm updates and modification. A preliminary examination of algorithm changes suggests future research in this direction. For example, by modifying inaccurate COPD ICD-9 coding, inappropriate exclusion of comorbidities, alternative ICD-9 coding for osteoarthritis and medication lists requiring updates, we found the PPV of COPD improved from 37% to 72% while maintaining sensitivity at 72%. Likewise sensitivity of osteoarthritis and depression improved to 76% and 84% respectively, with still adequate specificity at 92% and 95% respectively. Sensitivity for hypertension also improved to 81%. This magnitude of improvement demonstrates the importance of careful examination of contributing factors and accounting for those in ongoing efforts to maintain data quality and algorithm development.

### Strengths and weaknesses

The main strength of this study was its characterization of the source of discrepancy between the CPCSSN algorithm and chart review. This facilitates both the improvement of case detection in primary care EMR databases and provides the information necessary to pursue data quality improvement. It also provides users of the data with an indication of the kinds of limitations the data may have and allows them to identify whether further validation or verification may be necessary in order to utilize the data for a specified purpose. It is important to emphasize that this study focuses on internal validation and provides a reliable indication of the physician’s diagnosis and therefore sufficient for purposes where a primary care diagnosis is the variable of interest. [18] For uses requiring a high certainty that diagnoses are correct, external validation, which confirms the actual presence of a disease [18], using objective measures such as pulmonary function tests for COPD is necessary. However, the under-utilization of diagnostic tests such as spirometry in primary care [9,27,28] prevent this kind of validation from being performed exclusively using the medical record

The relatively small sample size cannot provide a comprehensive identification of all factors affecting misclassified or unidentified diagnoses in the database. Further, our sample was drawn from patients who were identified as having at least one of the target chronic conditions. While this increased the number of cases available for inclusion, there is limited representation of non-cases. However, the presented specificity and sensitivity values reflect relevant differences among the patient sample as a large majority had only 1 or 2 of the target conditions.

### Generalizability

Our primary objective was to explore factors that affect data quality for case finding algorithms. While the performance of computer based algorithms are limited to the CPCSSN algorithms used on MaPCReN data, the analysis of the underlying causes for discrepancy sheds light on key factors which may be generalized to other Primary Care EMR based databases around the globe. To the extent that we compared the audited patient record with the CPCSSN algorithms, we are confident the results are foundational for further examination of algorithm performance and improvement for those developing and evaluating primary care EMR databases.

## Conclusion

This study examines and describes the factors affecting the validity of diagnostic algorithms and data quality in a primary care EMR database. CPCSSN case finding algorithms applied to the EMR data were valid with a few important limitations. The algorithms showed limited sensitivity for COPD, depression and osteoarthritis, poor PPV for COPD and limited specificity for hypertension. Clinician and practice factors were associated with discordant diagnosis and data quality is challenged by the complexity of primary care encounters. This study provides insight into limitations and challenges for data quality in primary care EMR data and case definitions and further analyzed underlying causes for discrepancy. Several suggestions are made to immediately rectify deficits and facilitate ongoing improvement of algorithm performance and data quality in CPCSSN and other primary care EMR databases.

### Availability of supporting data

The data originated from 1) patients’ electronic medical records, and 2) region-specific data housed within the CPCSSN central repository. Neither are available in an open access data repository, however further information regarding accessing data within the CPCSSN database can be obtained from: http://cpcssn.ca/research-resources/cpcssn-data-for-research/

## Abbreviations

EMR: Electronic Medical Record
CPCSSN: Canadian Primary Care Sentinel Surveillance Network
MaPCReN: Manitoba Primary Care Research Network
HTN: Hypertension
DM: Diabetes mellitus
OA: Osteoarthritis
COPD: Chronic obstructive pulmonary disease
PBRN: Practice-based research networks
ICD-9: International Statistical Classification of Diseases and Related Health Problems Version 9
